# Synchronous Seasonal Change in Fin Whale Song in the North Pacific

**DOI:** 10.1371/journal.pone.0115678

**Published:** 2014-12-18

**Authors:** Erin M. Oleson, Ana Širović, Alexandra R. Bayless, John A. Hildebrand

**Affiliations:** 1 NOAA Fisheries, Pacific Islands Fisheries Science Center, Protected Species Division, Honolulu, Hawaii, United States of America; 2 Scripps Institution of Oceanography, University of California San Diego, La Jolla, California, United States of America; 3 Joint Institute for Marine and Atmospheric Research, University of Hawaii at Manoa, Honolulu, Hawaii, United States of America; Pacific Northwest National Laboratory, United States of America

## Abstract

Fin whale (*Balaenoptera physalus*) song consists of down-swept pulses arranged into stereotypic sequences that can be characterized according to the interval between successive pulses. As in blue (*B. musculus*) and humpback whales (*Megaptera novaeangliae*), these song sequences may be geographically distinct and may correlate with population boundaries in some regions. We measured inter-pulse intervals of fin whale songs within year-round acoustic datasets collected between 2000 and 2006 in three regions of the eastern North Pacific: Southern California, the Bering Sea, and Hawaii. A distinctive song type that was recorded in all three regions is characterized by singlet and doublet inter-pulse intervals that increase seasonally, then annually reset to the same shorter intervals at the beginning of each season. This song type was recorded in the Bering Sea and off Southern California from September through May and off Hawaii from December through April, with the song interval generally synchronized across all monitoring locations. The broad geographic and seasonal occurrence of this particular fin whale song type may represent a single population broadly distributed throughout the eastern Pacific with no clear seasonal migratory pattern. Previous studies attempting to infer population structure of fin whales in the North Pacific using synchronous individual song samples have been unsuccessful, likely because they did not account for the seasonal lengthening in song intervals observed here.

## Introduction

Song can be described as a regular and repeated sequence of sounds, organized into a hierarchical structure [1], although song complexity varies across taxa. For many species, song is associated with mate attraction, mate guarding, or territorial defense; however, precise song function remains unknown, particularly in baleen whales. Seasonal changes in song have been well studied in birds [2], and are also known to occur in baleen whales [3]. Humpback whale (*Megaptera novaeangliae*) songs evolve during the breeding season, but also change from one year to the next, with neighboring populations sharing some song components [4], [5]. In contrast, the temporal patterning of blue whale (*Balenoptera musculus*) songs remains relatively constant [6], even as the tonal frequency in song components decreases over time [3], [7]. Although not consistently referred to as song, the repeated regularly timed pulses that make up song have been documented from fin whales (*B. physalus*) in all ocean basins [8]–[11] and where populations have been monitored for extended periods, song structure has been relatively stable over time [9]–[11]. However, fin whales in a portion of the western North Atlantic have recently been shown to synchronously change the inter-pulse interval (IPI) of their songs rapidly, within about one month during the winter [12]. The prevalence and nature of seasonal change in fin whale song in other ocean basins has not been examined.

Fin whale songs consist of stereotypic sequences of down-swept pulses organized into regularly repeated patterns [8], [9], [13]. As in blue whales, these form a recognizable pattern in time (sensu [6]), even though the song is made up only of a single downswept unit type. The downsweeps, known as 20 Hz pulses [14], [15], are generally 1 s in duration and centered at approximately 20 Hz. In some regional variations, the pulse also includes a higher frequency component, which may be indicative of distinct populations [8], [9], [16], [17]. The 20 Hz pulses have been recorded from fin whales worldwide [10], [13], [18], and in the northern Pacific they occur most commonly during the fall and winter [19]. As with a number of other baleen whale songs, fin whale songs are thought to be produced exclusively by males [20].

Several baleen whale species exhibit geographic differences in vocalizations that may correspond to population boundaries. Minke and Bryde's whale call types vary across ocean basins [21]–[24] and at even finer scales that may reflect breaks in distribution [24]. In humpback whales, differences in songs among different populations have been well documented [25], though sharing of song elements among neighboring populations [4] may complicate attempts at acoustically identifying populations based solely on song. In blue whales, significant and geographically stable differences in the characteristics of song phrases have been proposed as a basis to delineate blue whale populations worldwide [6]. Some fin whale 20 Hz pulses are organized into distinct temporal patterns that also may help delineate their populations [8], [9], [17]. A doublet pulse, for instance, is an alternation between two different IPIs between successive down-swept pulses [11]. Using these patterns to determine stock boundaries, however, has proven to be complicated and works better for Atlantic populations [9], [26], than for the Pacific [9]. In particular, compilations of fin whale IPIs across the Atlantic have indicated a level of population structure, even when geographically dispersed samples came from different months or years [9]. In contrast, in the Pacific there is little concordance between geographically and temporally dispersed song samples [9]. While fin song IPIs in a particular region occurred in consistent patterns over a period of hours or days, these patterns generally do not align to suggest continuity among samples from different periods and regions across the broader North Pacific [9]. Only fin whale song recorded in the Gulf of California was consistent across a longer observation period [9] and was consistent with the song IPIs of a previously recognized resident population of fin whales [11]. These results suggest a better understanding of the seasonal patterns in the IPI of fin whale song is necessary before their use for population differentiation can be fully evaluated in the Pacific. Here we use long-term acoustic datasets collected over six years from Southern California, Hawaii, and the Bering Sea to examine the seasonality of fin whale song throughout the eastern North Pacific and evaluate how fin whale song in this region may be used to identify and track fin whale populations.

## Materials and Methods

**Figure 1:**
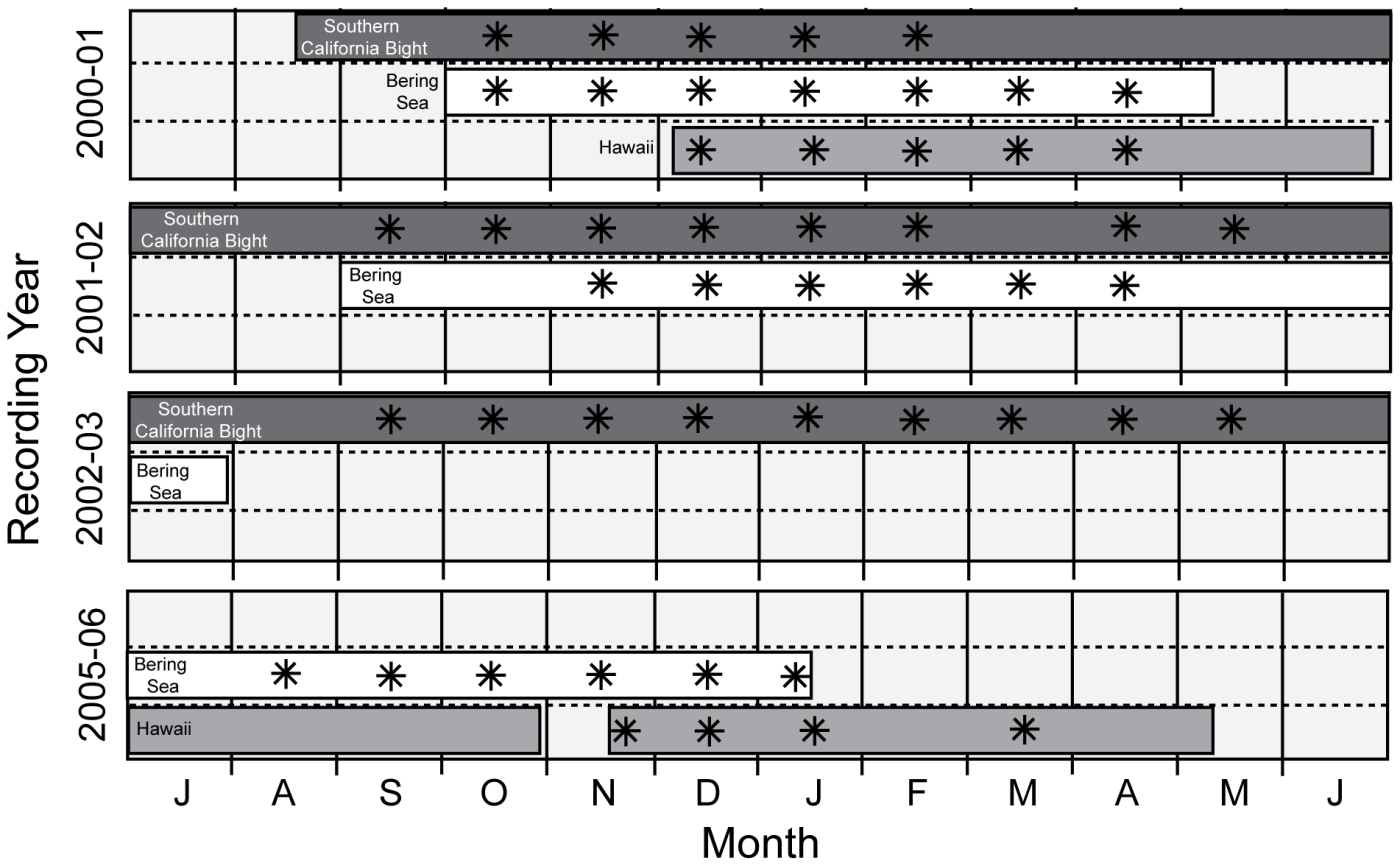
Acoustic data used for measurement of fin whale song and months with song present. Available data from each site are shown as bars by recording year. Years begin July 1 and end June 30 to encompass the continuous nature of fin whale singing throughout the winter season. Months with fin whale song heard during the days monitored at each site are indicated with an asterisk.

Two types of autonomous, seafloor-mounted passive acoustic recorders were deployed at sites off Southern California, in the Bering Sea, and near Hawaii between 2000 and 2003 and 2005–2006 (Table 1). Recorders were not deployed in protected areas or within waters requiring a permit from Federal or local agencies. From 2000 to 2003, Acoustic Recording Packages [27] or ARPs, capable of low-frequency (<1,000 Hz) monitoring were used in all three regions. The ARP data-logging system included a 16-bit A/D converter, 36 GB of storage capacity, a hydrophone tethered 10 m above the seafloor, a release system, ballast weights, and flotation. Data were recorded continuously with a sample rate of either 500 or 1,000 Hz, with a low-end roll-off of 5 Hz, resulting in an effective bandwidth between 5 and 250 Hz or 5 and 500 Hz, respectively. Off Southern California, ARPs were generally recovered and redeployed every 2 to 4 months, resulting in short (2 days or less) gaps in recording effort. Near Hawaii and in the Bering Sea ARPs were typically recovered and redeployed once per year, occasionally resulting in gaps in recording effort of several months (Fig. 1).

**Table 1:** Acoustic data used for measurement of fin whale song.

Region	location	year
Southern CA	32- 41.3N, 119- 01.9W	2000-01
	Same location	2001-02
	Same location	2002-03
Bering Sea	57- 00.0N, 164-59.97W	2000-01
	56- 49.9N, 163-00.5W	2001-02
	56-51.02N, 164-03.52W	2005-06
Hawaii	19- 07.97N, 155-04.76W	2000-01
	18-43.33N, 158-15.23W	2005-06*

Years begin July 1 and end June 30 to encompass the continuous nature of singing within that period. * Duty-cycled recording: 5 min on during every 25 min.

Beginning in 2005, upgrades to the acoustic system allowed for high-frequency recording by High-Frequency Acoustic Recording Packages [28], or HARPs, near Hawaii and in the Bering Sea. HARPs record acoustic data using a 16-bit A/D converter onto 16 drives totaling 2 TB of storage capacity. Off Hawaii, the HARP was deployed in similar fashion to previous ARPs, with the hydrophone tethered approximately 10 m above the seafloor. Data were recorded at Hawaii at 200 kHz sample rate. Due to the high data rate and limited data storage capacity, and desire to monitor as much of a full year as possible, HARP data collected near Hawaii were duty-cycled, such that the HARP recorded for 5 minutes, then was off for 20 minutes. In the Bering Sea, the HARP was integrated into a Pacific Marine Environmental Laboratory (PMEL) mooring, such that the hydrophone was 5–10 m off the seafloor at approximately 70 m depth. HARP data recorded in the Bering Sea was not duty-cycled, but was recorded at a lower data rate of 40 kHz to allow for year-round data collection. All high-frequency acoustic data were decimated to 2,000 Hz sample rate for this study, resulting in an effective bandwidth of 10 to 1,000 Hz.

Acoustic data were analyzed using custom-written software *Triton* [28] within MATLAB. For each year of data used for this study, one or two full days per month were randomly selected to be manually examined for song and a single analyst marked fin whale song pulses for that entire dataset. To minimize variation in the analyst ability to accurately define individual fin whale pulses within the spectrogram, we standardized viewing window parameters (size of the window, duration shown in each window, visible bandwidth, contrast and brightness of the displayed signal) and the analysis parameters (FFT length and overlap) to ensure consistent time and frequency resolution across datasets. Data were viewed as spectrograms, 120 s per screen from 0 to 100 Hz, in 1 Hz bins with 80% temporal overlap,. When a single singing fin whale, i.e. a fin whale producing stereotypic sequences of 20 Hz pulses in regularly repeated pattern, could be identified within the acoustic data, the start time of each pulse was manually noted and saved for later analysis using a call logger within *Triton*. All pulses within an individual song sequence were marked and only song sequences of at least 2 minutes duration were used for analysis. Occasionally more than one whale could be heard singing at once, identifiable as overlapping sequences of regularly repeated pulse patterns. In this case pulses from each individual were marked in series, with all song pulses from one sequence marked, followed by all song pulses from the overlapping sequence. If it became difficult to distinguish individual sequences from each other, no additional pulses were marked. It is probable that song segments were measured from more than one whale per day given the occurrence of overlapping songs or long (i.e. several hour) breaks in song on a given day. Pulses that were not clearly part of song sequences were not marked or enumerated during this analysis. Because more than one analyst contributed to song IPI measurements over the whole dataset, inter-analyst variability was examined by assigning each of the three contributing analysts to mark the same sequence of song pulses over 4 separate one-hour periods selected from the entire song dataset. Differences in measured IPIs between analyst pairs were compared and found to have an overall average absolute difference in IPI of less than 0.1 s with standard deviation 0.33 s.

**Figure 2:**
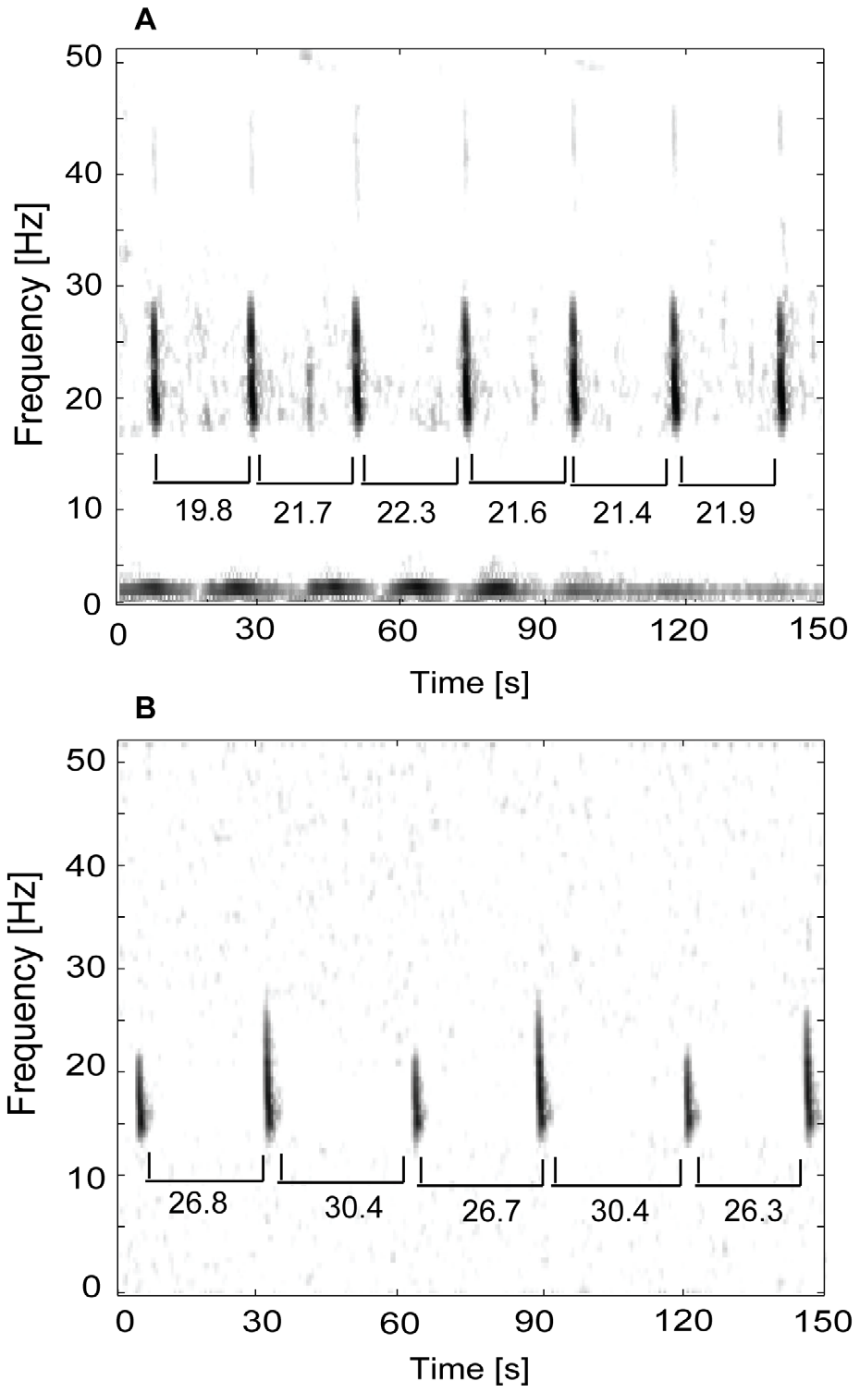
Spectrograms of fin whale singlet and doublet song. The IPI (s) is shown below the pulses for each sequence. Panel A shows a song sequence with a single IPI recorded off Southern California in October 2002 and Panel B shows a song sequence with doublet IPIs from Hawaii in December 2002. Note that the timing of singlet IPIs is more variable than doublet IPIs. Spectrogram characteristics: 1-s FFT, 80% overlap. Occassionally the short IPI of the doublet pair is repeated between doublet sequences, most commonly off Southern California (not shown).

Inter-pulse intervals were calculated using the start time of each pulse within an individual song sequence (Fig. 2). No attempt was made to attribute song sequences to a specific individual singer across breaks in song or across artificial breaks created by duty-cycling. For this reason we are unable to measure song duration with confidence. The time-series of daily IPIs were examined to ensure that they formed a consistent patterned sequence. A typical doublet song is defined as a sequence consisting of a short IPI followed by a long IPI (Fig. 2B), with this pattern repeated for at least several minutes. Song variants consisting of only a single IPI or including mixtures of doublet and singlet intervals were also observed. In the case of singlet series, IPIs were consistently equivalent to the short IPI in doublet song. These variations were not treated as separate songs nor separated in later analysis because inspection of IPI time-series suggested these song mixtures were variant combinations of the same short and long IPIs that comprise doublets. The number of short and long IPIs marked in each day was also enumerated.

Monthly variation in fin whale song was assessed by first calculating monthly median short and long IPIs. Variability within song IPIs within each month and site was compared using Median Average Deviation (MAD). The MAD is the median of the absolute deviations from the data's median and is a robust measure of statistical dispersion, more resilient to outliers in a dataset than the standard deviation [29]. For a symmetric distribution, the MAD is equivalent to the 75^th^ percentile of the distribution. The 25% and 75% quartiles of daily IPIs were also calculated to evaluate variability within song.

Following inspection of monthly IPIs and identification of gradual change between October and February across all three sites, a median IPI was calculated for October, December, and February at each site for each year with data in those months. We define the start of the song year to be July 1, as there is typically a break in the occurrence of fin whale song during the summer at all sites. These monthly measures represent median IPI lengths for early, middle, and late season song, providing quantitative measures of IPI length at different times during the year and facilitating comparison with future studies of fin whale song that may have access to shorter periods of data. The average monthly change in median IPIs was calculated from October to February, or from the first to last month with song within that period, to compare seasonal rate of change across monitoring locations.

When we found substantial variability around the median IPIs in some months (assessed as first or third quartiles greater than 1), we further assessed the data to determine whether this variability could be attributed to plasticity in IPIs of the same song type or if the variability could represent occurrence of more than one song type. The distribution of song IPIs was evaluated for each month with high variability using histograms to examine whether IPIs clustered into a single short and long IPI peak or might be separated into multiple song peaks.

No protected species were invasively sampled during the study.

## Results

**Figure 3:**
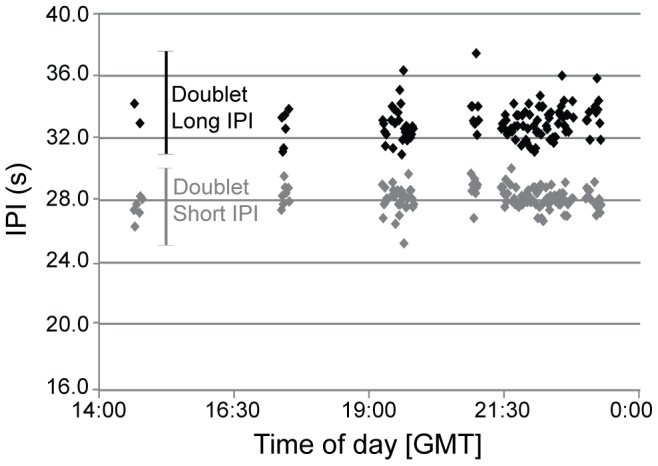
Example examination of fin whale doublet song IPIs. All fin whale song IPIs recorded at Hawaii on February 19, 2001. Long (black) and short (gray) IPIs are shown as marked by the anlayst. See Fig. 2 for timing and frequency differences between short and long IPIs in spectrogram form.

Fin whale song pulses were marked for three years of acoustic data recorded off Southern California and in the Bering Sea, and two years of data recorded near Hawaii, resulting in over 25,600 marked song pulses (Table 2). The average number of song pulses marked per day across all sites was 282, though this varied seasonally and among sites with a low of 74 pulses in August 2005 in the Bering Sea and a high of 564 pulses in April 2001 off Hawaii. Songs in all three regions most commonly consisted of a doublet pattern, with consecutive pulses occurring at alternating shorter and longer IPIs (Figs. 2 and 3), with this pattern repeated for up to several hours. Off Southern California, song sequences occasionally included additional short IPIs between doublet IPI pairs, producing variants on the standard doublet song. Although these additional short IPIs resulted in variability in the ratio between short and long IPIs within a measured song sequence (Table 2), the additional short IPIs appear consistent with that typically measured between consecutive doublets and therefore were not treated as a different song type. Less frequently, across all three regions, singlet song sequences consisting only of the shorter IPI from the doublet sequence were also heard. Sequences of doublets were occasionally followed or preceded by sequences with only shorter interval pulses.

**Table 2:** Seasonal characteristics of fin whale song.

region	year	months with song	marked calls	October median IPI (s)	December median IPI (s)	February median IPI (s)	Monthly Change in IPI (s)
Southern CA	2000–01	October–February	1486/935	20.7 (0.6)/26.6 (0.8)	23.5 (0.6)/29.6 (0.8)	25.7 (1.0)/32.3 (1.1)	1.4/1.7
	2001–02	October–May	2255/740	18.0 (0.5)/24.3 (1.1)	23.5 (0.9)/30.0 (0.8)	27.9 (0.6)/33.2 (1.1)	1.6/1.5
	2002–03	September–May	2984/1676	20.5 (0.6)/26.3 (0.8)	23.8 (0.5)/30.1 (0.6)	27.9 (0.8)/32.6 (1.3)	1.8/1.5
Bering Sea	2000–01	October–April	2797/2605	21.3 (0.5)/—	26.4 (0.5)/31.7 (0.4)	29.2 (0.3)/34.3 (0.6)	2.2/2.1
	2001–02	November–April	2720/2368	—	22.9 (0.7)/28.5 (0.7)	28.7 (0.3)/33.5 (0.4)	2.1/2.0
	2005–06	August–January	1533/1478	23.5 (0.4)/28.4 (0.4)	25.1 (0.5)/30.1 (0.7)	—	1.7/1.6
Hawaii	2000–01	December–April	1450/1077	—	22.2 (0.9)/27.5 (1.1)	28.7 (0.5)/34.2 (0.8)	2.9/2.7
	2005–06	November–March*	162/152	—	26.6 (0.7)/31.5 (1.0)	—	2.0/1.6

The total number of marked calls, as well as the October, December, and February song IPIs and median absolute deviation (in parentheses) are presented, with short IPIs shown before the/and long IPIs after. IPIs from song segments consisting of only short IPIs or with an intermittent series of short IPIs (mixed IPI songs) are included in calculation of short IPI for each month. Monthly change in IPI was calculated from October, or the first month with song if later, to February, or the last month with song if earlier.

* Although fin whale song was recorded in March of 2006, no song was evident in February, 2006.

**Figure 4:**
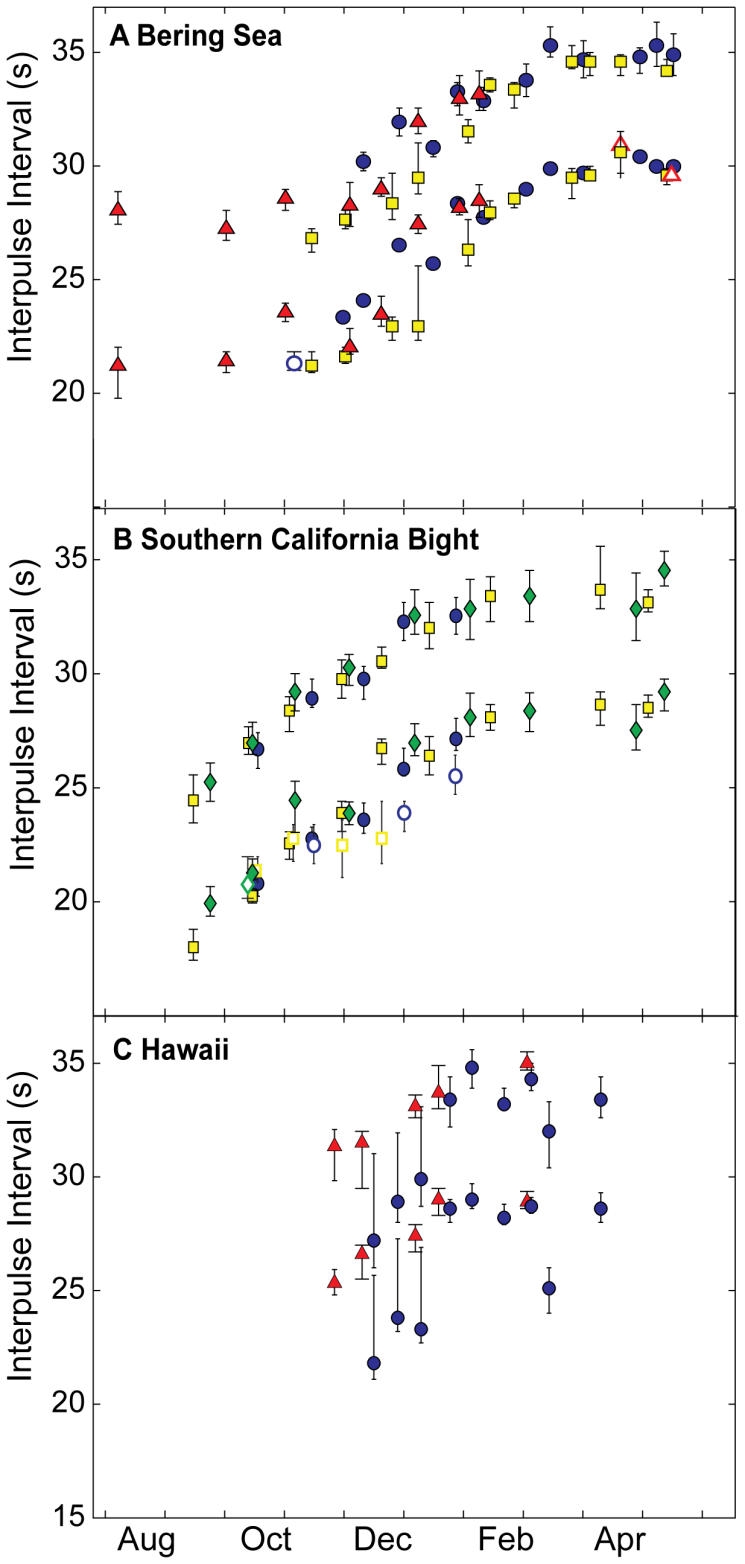
Median and 25% and 75% quartiles of fin whale interpulse intervals (IPI) by region. Panel A shows fin whale IPIs from the Bering Sea, panel B from Southern California, and panel C from Hawaii. Both the short and long IPIs within a doublet pair are shown for each month unless only one interval was measured within a given month and site. Across all three panels each singing year is designated by a different symbol and color: 2000-01 —blue circles, 2001-02 — yellow squares, 2002-03 — green diamonds, and 2005-06 — red triangles. Open symbols represent months when singlet IPIs were also detected and measured. Location and recording details in Table 1.

Doublet song was first recorded in August or September and continued through April or May in the Bering Sea and off Southern California. Similar doublet song intervals were present in all three regions from November or December to March or April of each year (Table 2). At all sites, IPIs lengthened seasonally, beginning the season with a consistently shorter and ending the season with consistently longer set of IPIs (Table 2, Fig. 4). Song IPIs gradually increased at all three sites from October to March, then leveled off at relatively longer IPIs until singing was no longer heard or data collection ended at each site. Early season (August to October) singing in the Bering Sea also occurred with consistent short doublet IPIs. The monthly change in IPI from October to February, encompassing the period of greatest change across all sites, was fairly consistent with an average rate of increase of 2.0 s and 1.8 s per month (Table 2), respectively for the short and long IPIs present in the song. Song intervals annually reset to roughly the same IPIs in the late-summer or fall of each year, generally following a period when no song was detected at each site. The rate of change was more variable in Hawaii in 2000, where limited data and periods with two possible song types (see below) likely contributed to that variation.

**Figure 5:**
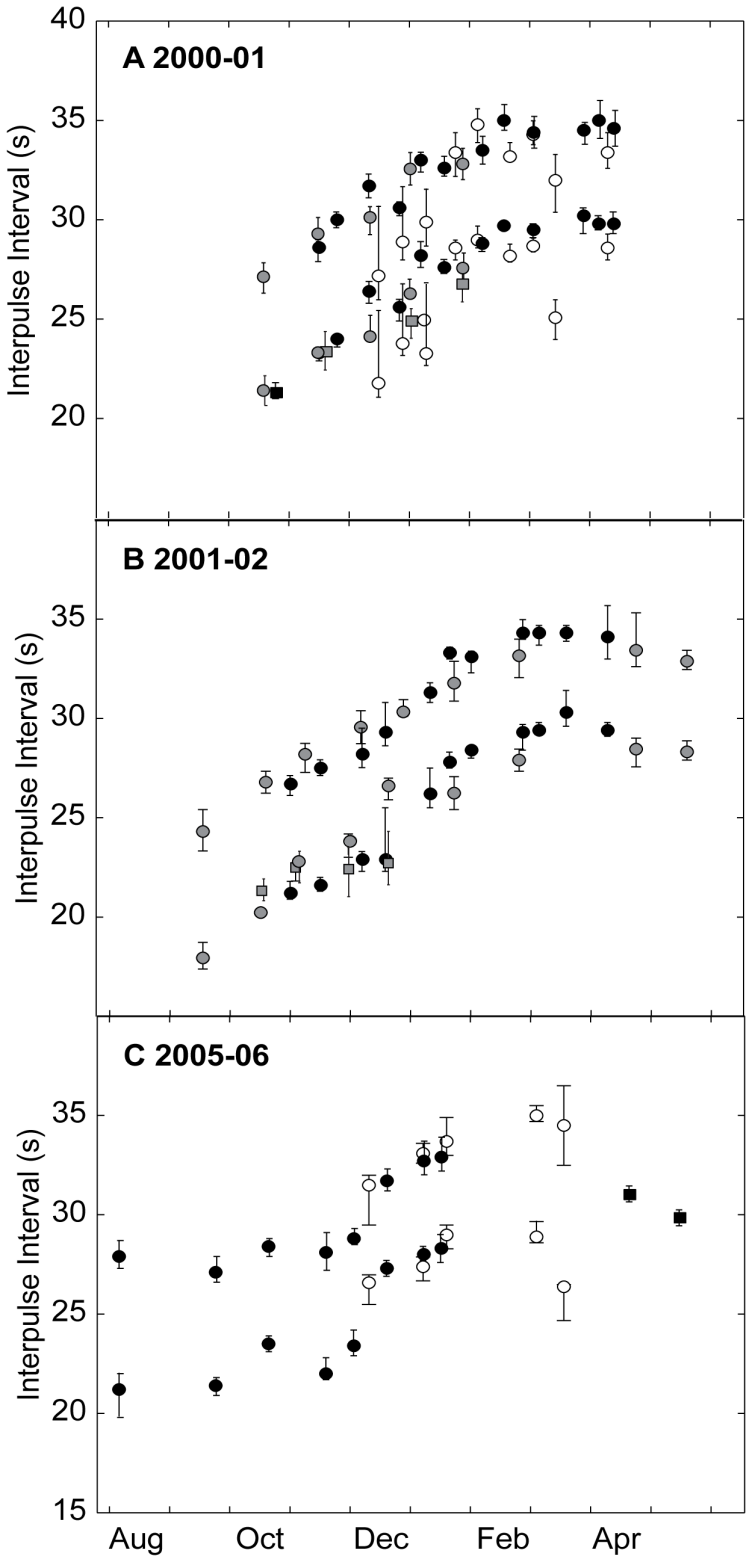
Median and 25% and 75% quartiles of fin whale interpulse intervals (IPI) by year. Panel A shows fin whale IPIs from 2000-01, panel B from 2001-02, and panel C from 2005-06. Consistent across all panels, fin whale IPIs from the Bering Sea are shown in black, off Southern California in gray, and near Hawaii in white. Short and long IPIs within a doublet pair are shown for each month as circles. Squares represent months when singlet IPIs were also detected and measured. Location and recording details in Table 1.

When fin whale songs were recorded during the same season at two or more sites- 2000-01 at all three regions, 2001-02 off Southern California and in the Bering Sea, and 2005-06 off Hawaii and in the Bering Sea- IPIs were similar across sites, though some inter-annual and geographic variability was evident (Fig. 5, Table 2). In particular, IPIs appeared to have tighter correspondence between regions in 2001-02 and 2005-06 than during 2000-01 (Fig. 5), indicating that the degree of synchrony between fin whales across this large region varies annually.

**Figure 6:**
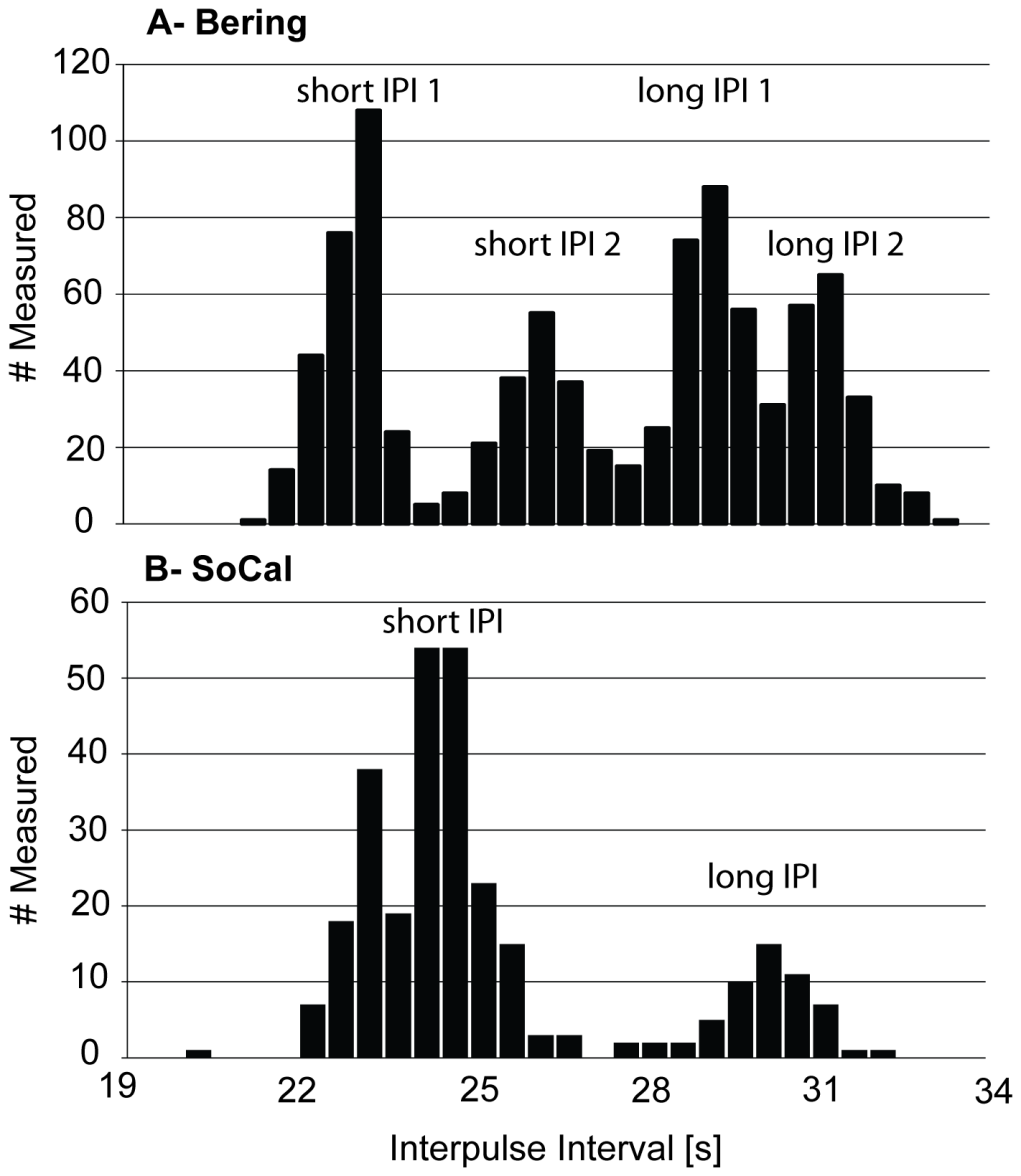
Relative occurrence of IPIs in December. Higher variability in song IPIs in December at some sites (see Table 2) is partially explained by the occurrence of two separate song types with IPIs offset by several seconds. Panel A shows IPIs for December 2001 in the Bering Sea, panel B for December 2001 off Southern California. This presentation of all IPIs measured on a single day illustrates that the song measured for the Bering Sea in December 2001 is composed of two different songs, one with relatively shorter short and long IPIs (labeled ‘short IPI 1’ and ‘long IPI 1’) and another with relatively longer IPIs (labeled ‘short IPI 2’ and ‘long IPI 2’). Whether these variations represent variability within a single singing whale or the presence of two or more whales singing different song on the same day cannot be assessed with the data we have available.

While variability within each marked day was generally low (0.3–1.3 s MAD, Table 2), IPIs in December, roughly the middle of the song season at all sites, showed the greatest amount of variability within and between sites (Fig. 4). Post-hoc examination of the distribution of IPIs in December revealed variability in the song patterns at all sites (Fig. 6). In the Bering Sea and near Hawaii it appears that two different song types occurred on the same day, one with shorter IPIs than the other. This is supported by visual inspection of IPI time-series for these sites, where periods of silence of an hour or more separated periods with different doublet intervals. In contrast, IPIs from Southern California showed relatively little variability in IPI during that period, but a higher proportion of short versus long IPIs within the doublet series (Fig. 6B). In general, fin whale songs were less precisely synchronized within or across sites in December, the mid-point of the singing season, than during the rest of the singing period.

## Discussion

Fin whale song exhibits a synchronized pattern of change in the IPIs across the eastern North Pacific. In three regions and across four years, fin whale singlet and doublet song sequences had IPIs that gradually increased during the singing season at a rate of ∼2 seconds/month from October to February. Our finding of similar IPI structure across three regions in the eastern North Pacific, particularly within the context of synchronous seasonal change, suggests connectivity of the fin whale population throughout this region. The pattern of fin whale song in the Pacific is different from the seasonal trends in fin whale song in the Atlantic. In the Atlantic, fin whale song IPIs transitions over the course of one month from singlet “short” (9.6 s) IPI (also referred to as inter-note interval or INI) present September through January to singlet “long” (15.1 s) IPIs present March through May [12], resulting in a more step-like change, rather than the gradual change in doublet timing that we observed from October to February. In the Pacific, the IPI reset annually and was fairly synchronous across the region, though variability in IPIs was evident at all sites. IPIs also reset annually in the Atlantic, and there may be an increase in IPI from year to year [12].

Although variability in the IPI was generally low within months at individual sites, as well as across sites and years, there were a few exceptions. Variability was particularly high in December of each year, roughly the mid-point of the singing season. In those months, it appeared that not all fin whale songs sampled on a given day consisted of the same IPIs (for example, see Fig. 6). In 2000-01 in the Bering Sea and off Hawaii, the occurrence of two similar, but not identical patterns of IPI on the same day significantly contributed to the variability in median IPI. The proportion of short versus long IPIs within doublet pairs also varied. Deviation from the doublet pattern generally arose through the addition of or exclusive use of a single short IPI consistent with the shorter IPI in the doublet pair. Fin whales off southern California, in particular, seemed to produce a much higher proportion of single IPIs than in the other two regions (Table 2). Variation in the use of singlet versus doublet IPIs, and slight variations in IPIs at the same time across sites suggests behavioral plasticity in song sequencing among and between individual fin whales.

### Geographic variation and population structure

Unlike the song units produced by other baleen whales, fin whale 20 Hz pulses have been recorded in all ocean basins, without any consistent differences in the frequency or temporal characteristics of the pulses themselves between fin whale populations or oceans. However, different IPIs have been shown to correspond to putative fin whale populations in the Atlantic [7] and Mediterranean [8]. Although fin whales in the Gulf of California are known to produce a distinct doublet pattern [11], much shorter than those observed here, attempts to find structure in fin whale song recorded at different times and places in the Pacific have proven difficult, without clear geographic patterns emerging [7]. Our result that fin whale song changes seasonally offers an explanation for the lack of clarity in previous studies. Within the eastern North Pacific, examination of fin whale population structure via song IPI must account for the seasonal variability in IPIs observed here. Simple comparisons of IPIs measured at different times and locations throughout the region are not likely to find any coherent pattern.

The traditional view of baleen whale life history is that whales migrate from high latitude feeding grounds in the summer to lower latitude calving grounds in the winter [30]. We recorded fin whale calls at both high and low latitude areas during the fall, winter, and spring. Our recordings may suggest that fin whale seasonal movements are more nuanced and the migration is not as comprehensive as the traditional theory suggests. A more complex pattern of movement across the eastern North Pacific was also indicated from historic records, such as the whaling data and mark recovery tags, and from more recent visual observations and other acoustic recordings [31], [32]. The synchrony in calling across the basin additionally suggests there may be a constant exchange of information across a large part of the eastern North Pacific Ocean during most of the year, suggesting more continuous movement of individuals among these regions rather than discrete seasonal migrations. However, the variation in fin whale song IPIs in the Bering Sea and near Hawaii, particularly the occurrence of two doublet song types with similar but distinctly different IPIs on the same day, may be indicative of an overlap with another central or western Pacific population at the edges of the population range. Two stocks of fin whales, North American and Asian, have been previously suggested [33] and may thus be further supported by these data.

### Analysis considerations and limitations

In addition to population structure and migration patterns, our results also have a practical implication on the use of passive acoustics for fin whale monitoring. Passive acoustic data have been used to describe seasonal and annual distribution and relative abundance of fin whales worldwide [10], [13], [19], [34]. While those results are usually framed with appropriate caveats, our results caution against using pure call counts as a measure of relative abundance, as the number of calls a whale produces in an hour will intrinsically change with the change in the IPI. Interestingly, it does not appear that the maximum number of calls were detected in the Atlantic or the Pacific during the time of the year with the highest singing rates (i.e. lowest IPI), late summer and early fall [12], [19], indicating a complex relationship between call production rates and animal abundance. Seasonal variability in the occurrence of song must be accounted for, in addition to addressing seasonal changes in IPI within a population, before call counts can be used as a proxy for abundance.

Limitations within our own analysis of fin whale song are also important to acknowledge. Most notably, due to the inability to localize vocal individuals or track calling sequences across breaks in song or across periods without data collection (i.e. duty-cycling), we cannot account for the variation due to the number of callers sampled in each day, or the duration of singing by an individual. This limitation could be important if a single animal is more likely to maintain a consistently timed song, and variation in aggregate IPIs was directly related to the number of measured singers. An additional limitation of the presented analysis is also the use of human analysts to manually mark the start time of individual song pulses. We attempted to minimize variation in the ability to accurately identify the start of individual calls within the spectrogram by standardizing viewing window parameters (size of the window, duration shown in each window, visible bandwidth, contrast and brightness of the displayed signal) and the analysis parameters (FFT length, overlap), and also looked for differences in measured IPIs of the same song sequences across analysts. Although some variation within and among analysts is present, the median difference in a subset of song IPIs measured by all analysts indicates they were marking song pulses in a consistent manner with high precision (median difference <0.1 s, SD = 0.33 s), the seasonal and annual patterns observed here cannot be attributed to error by the human analysts. Further, the level of variation observed within each day is fairly low (0.3–1.3 MAD) in most cases and does not obscure the overall patterns of individual songs, nor the seasonal change in song IPIs. While use of energy-based peak detectors may result in more accurate selection of pulse start times, analyst consistency was high and any variation due to the accuracy of analyst picks is overwhelmed by the overall song patterns. Finally, because songs were marked on only two days per month, overall variation in fin whale calling at each site is likely underestimated, with some song variants potentially missed. The additional variation may be significant when more singing whales are in or passing through the region at the peak of the singing season.

### Song synchrony and the role of song

Despite some of these uncertainties, the occurrence of consistent annual change in fin whale song IPIs within and across monitoring sites over six years raise the question- why is there a strong circannual rhythm in fin whale song patterns? Circannual rhythms are common in mammals and birds [35], [36] and can be linked to endogenous, reproduction-related hormonal activity [37], [38]. Little is known about the mating behavior of fin whales [39], but it is assumed the songs have a mating function [20]. The timing of the change in the song IPI seems to coincide with the fin whale mating period in the North Pacific and North Atlantic, where the mating continues from November to March [33] and peak conception is estimated to occur in December [40]. Male fin whales also have an annual cycle in testes activity, which peaks during peak conception [41]. This coincidence in the timing of changes in the IPI of the male song in relation to peak reproductive activity suggests that the two may be linked.

Male signaling during the reproductive season can serve a range of purposes: to guard territory or for resource advertisement, to facilitate female choice, or in male-male interaction [42]. While evaluation of song IPIs alone certainly provides an incomplete view of song function, some potential functions of song may be evaluated within the context of our observations. Since the three regions with synchronous singing span from the oligotrophic Hawaiian waters to the highly productive sub-Arctic waters, resource advertisement or guarding is probably not a driving factor for the singing displays. Similar to blue whales, the production of low frequency, high amplitude calls is likely energetically expensive [43] and it may not be possible to sing and feed concurrently [44], so singing may be a proxy for male fitness and could promote female choice. Singing at a faster rate early in the breeding season may indicate high male fitness and promote pairing between males and females. Singing rates appear to be related to pair bonding in some bird species [45] with a decrease in singing rate following pairing. In blue whales, pairs often persist into the breeding season [46]. If the same model applies to fin whales, the singing rate may be related to the prevalence of mating pairs, with the singing rate declining (IPI increasing) as the mating season progresses.

An alternative driver of this behavior, rather than attraction of females, could be male-male interactions, either antagonistic, such as mate guarding or direct competition, or social communication. The decline in the call rate during peak conception (November to March) observed in this study is not consistent with the mate guarding hypothesis, which predicts peak call rates during peak conception [42]. If the song functions as an intrasexual competitive display, however, the slower rate of calling through the breeding season could indicate a decrease in male-male competition over the course of the breeding season. In addition, synchrony in calling could be maintained as the result of male-male communication, where the singing male's response matches the call of the neighbor to promote communication [47]. Even among humpback whales, the baleen whale species with the best studied social and breeding system, studies have suggested both an intersexual [48], [49] and intrasexual role [50], [51] in song production for that species. Without more information on the fin whale breeding system, it is not possible to evaluate the likelihood of the male-female versus male-male interactions as dominant drivers of this behavior.

## Conclusions

Passive acoustic data concurrently collected during four years at three different locations across the eastern North Pacific have revealed synchronous seasonal change in fin whale song across this broad region. The song IPIs reset annually to a relatively short singlet or doublet in the late summer and fall and progress to longer IPIs by the spring in the Bering Sea, near Hawaii, and off southern California. Such changes are difficult to interpret given the paucity of information on fin whale social structure and mating systems, but do suggest connectivity of fin whales across the eastern North Pacific. Additional study will be required to determine how wide-spread these seasonally changing IPIs occur, whether they truly represent a single population, and whether and how well broad sampling can be used to infer population movements, connectivity, and ecology over the long-term.
